# SOSAS Study in Rural India: Using Accredited Social Health Activists as Enumerators

**DOI:** 10.5334/aogh.2340

**Published:** 2019-03-14

**Authors:** Srivarshini E. Cherukupalli, Manisha B. Bhatia, Marissa A. Boeck, Kevin J. Blair, Neeraja Nagarajan, Shailvi Gupta, Leah C. Tatebe, Sristi Sharma, Ashish Bhalla, Benedict C. Nwomeh, Mamta Swaroop

**Affiliations:** 1 Northwestern University Feinberg School of Medicine, Department of Surgery, Chicago, US; 2 Texas Tech University Health Sciences Center, Lubbock, US; 3 Brigham and Women’s Hospital, Center for Surgery and Public Health, Boston, US; 4 New York Presbyterian Hospital, Columbia University Medical Center, Department of Surgery, New York, US; 5 University of California Los Angeles, Department of Surgery, Los Angeles, US; 6 Johns Hopkins University School of Medicine, Department of Surgery, Baltimore, US; 7 Brigham And Women’s Hospital, Department of Surgery, Boston, US; 8 University of California San Francisco, East Bay, Department of Surgery, Oakland, US; 9 Surgeons Overseas, New York, US; 10 Postgraduate Institute of Medical Education and Research, Chandigarh, IN; 11 Ohio State University, Nationwide Children’s Hospital, Department of Pediatric Surgery, Columbus, US

## Abstract

**Background:**

Global estimates show five billion people lack access to safe, quality, and timely surgical care. The wealthiest third of the world’s population receives approximately 73.6% of the world’s total surgical procedures while the poorest third receives only 3.5%. This pilot study aimed to assess the local burden of surgical disease in a rural region of India through the Surgeons OverSeas Assessment of Surgical Need (SOSAS) survey and the feasibility of using Accredited Social Health Activists (ASHAs) as enumerators.

**Material and Methods:**

Data were collected in June and July 2015 in Nanakpur, Haryana from 50 households with the support of Indian community health workers, known as ASHAs. The head of household provided demographic data; two household members provided personal surgical histories. *Current surgical need* was defined as a self-reported surgical problem present at the time of the interview, and *unmet surgical need* as a surgical problem in which the respondent did not access care.

**Results:**

One hundred percent of selected households participated, totaling 93 individuals. Twenty-eight people (30.1%; 95% CI 21.0–40.5) indicated they had a current surgical need in the following body regions: 2 face, 1 chest/breast, 1 back, 3 abdomen, 4 groin/genitalia, and 17 extremities. Six individuals had an unmet surgical need (6.5%; 95% CI 2.45%–13.5%).

**Conclusions:**

This pilot study in Nanakpur is the first implementation of the SOSAS survey in India and suggests a significant burden of surgical disease. The feasibility of employing ASHAs to administer the survey is demonstrated, providing a potential use of the ASHA program for a future countrywide survey. These data are useful preliminary evidence that emphasize the need to further evaluate interventions for strengthening surgical systems in rural India.

## Background

Global estimates show an astounding five billion of the world’s seven billion people lack access to safe, quality, and timely surgical and anesthesia care [1,2]. Furthermore, the number of surgeries that do occur in low- and middle-income countries (LMICs) is much lower than that of high-income nations [1,2]. The poorest third of the world’s population in LMICs receives only 3.5% of the surgical operations performed worldwide [3]. Until recently surgical care in LMICs was largely overlooked, with global health attention focusing instead on communicable diseases, and maternal and infant mortality [4]. However, an ongoing epidemiological transition to non-communicable diseases in LMICs, such as cancer, cardiovascular disease, and road traffic injuries, is increasing the focus on surgical care [2]. Surgical and anesthetic services are now being prioritized as an essential component of health care in LMICs [5]. Investing in surgery also has significant economic and welfare benefits, as untreated surgical conditions increase medical costs, disability, and death [2].

In order to facilitate the development of LMIC health care systems that include surgical care, it is necessary to understand the local burden of surgical disease. Modeled data has traditionally been used to estimate rates of surgery using sources such as governmental agencies, statistical organizations, and published studies [3]. However, the bulk of the published data are derived from hospital registries, which have limited epidemiologic value. In contrast, surveys completed at the community level provide a comprehensive measure of real-time surgical need [2,6]. The most widely used population-based tool to estimate burden of surgical diseases is the Surgeons OverSeas Assessment of Surgical Need (SOSAS) survey, which has been previously utilized in Sierra Leone, Rwanda, Nepal, and Uganda [6,7,8,9,10,11,12,13].

Several studies have been published describing surgical or trauma care capacity at facilities in India, but via a review of the literature, no community-based survey tool has yet been utilized to determine India’s surgical disease burden [14,15,16]. India, one of the largest countries in Asia with a population of 1.2 billion, remains a lower-middle income country with a Gross National Income (GNI) that falls below the average GNI of other lower-middle income countries (1,600 USD vs. 2,032 USD) [17]. Over 20% of the population lives below the national poverty line, and nearly 70% lives in rural areas, where access to health care is poor [17,18,19,20]. Government health centers in remote regions frequently have inadequate resources, and doctors regularly do not meet the necessary education and training criteria for their positions [20]. Furthermore, limited access to higher-level hospitals leaves patients with more complex conditions traveling greater distances to obtain the care they need [21,22].

In 2005, India launched the Accredited Social Health Activist (ASHA) program to address the challenges of limited access to health care facilities and insufficient infrastructure to effectively treat the rural population. One ASHA is trained as a community health worker for every 1000 people in rural India. Nearly 850,000 ASHAs across India currently provide antenatal and postnatal care, family planning awareness, sanitation education, nutritional supplements, and patient referrals to local health centers [23,24].

A household needs assessment performed in 2012 demonstrated that there were significant socioeconomic disparities within Nanakpur, a rural community in Haryana, India [25]. The objective of this pilot study was to assess the burden of surgical disease in a representative rural area of India using the SOSAS survey, the results of which would serve as preliminary evidence towards the need to further strengthen surgical systems in India. Importantly, the study also aimed to determine the feasibility of using ASHAs to conduct the survey in the hopes of developing a reliable methodology for countrywide survey deployment.

## Methods

Over two weeks between June and July 2015 data were gathered in Nanakpur, Haryana. Study approval was obtained from Northwestern University’s Institutional Review Board and the Director General Health Services of Panchkula, Haryana.

### Site Selection

**Figure 1:**
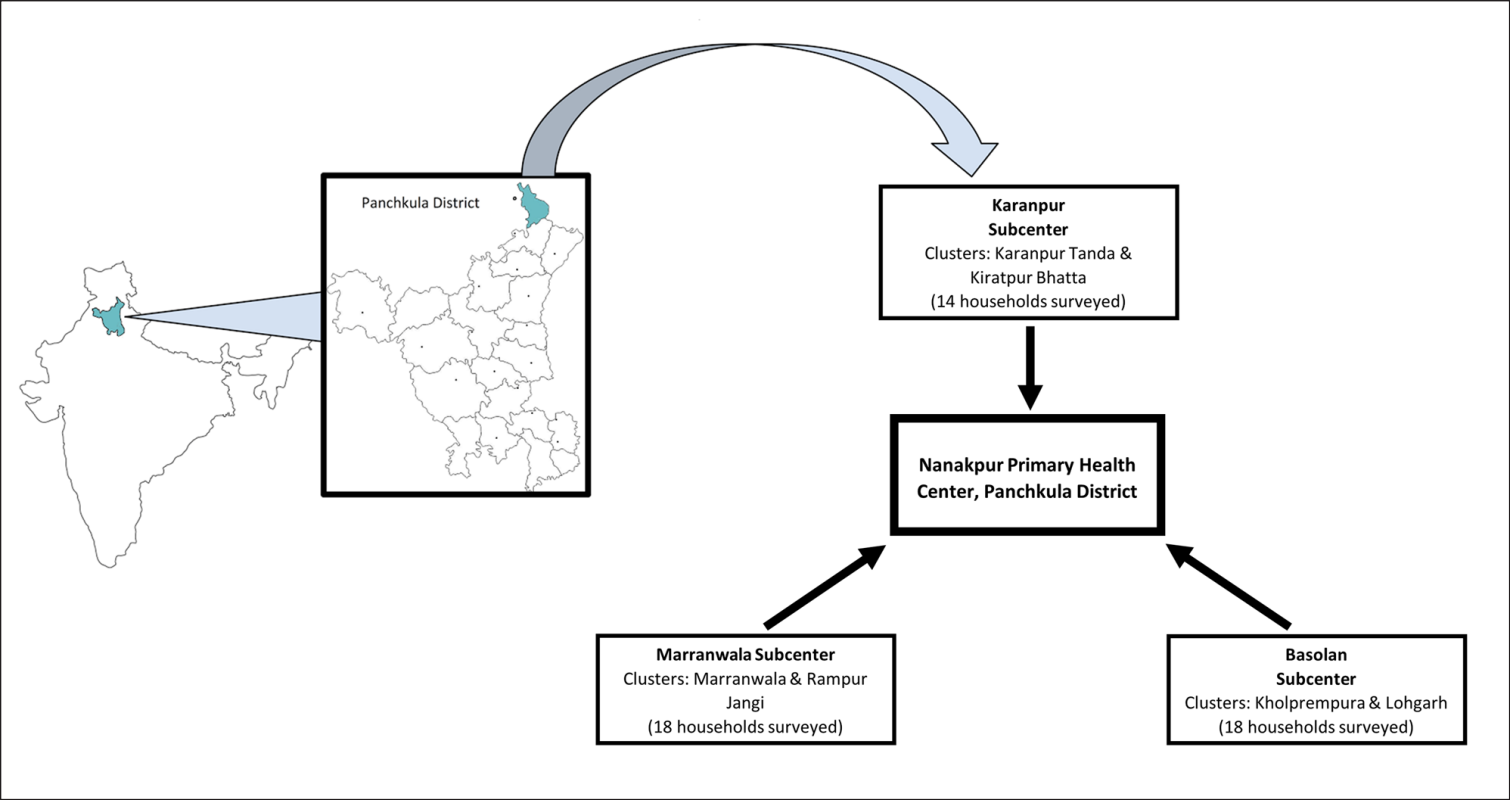
Location of Panchkula District and Nanakpur’s three subcenters. Clusters interviewed in each subcenter are listed, as well as the number of households surveyed.

Haryana is one of 29 states in India with a population of 2.53 million [26]. Nanakpur is a rural collection of villages located in the Panchkula district, which is one of 22 districts in Haryana (Figure 1) [27]. Nanakpur’s borders are defined by the area served by Nanakpur’s Primary Health Center; it is approximately one-tenth of Panchkula’s 315 mi^2^ area. The community of Nanakpur has a population of 37,168, with its large farming and migrant brick manufacturing workforce being representative of much of India’s rural population [25,28] A longstanding relationship between the authors and staff at Nanakpur’s Primary Health Center allowed for easy coordination of study logistics [25].

### Survey Instrument

The SOSAS tool consists of two sections: the first collects demographic information from the head of the household, including age and sex of household members; time, distance, and cost of traveling to the nearest primary, secondary, and tertiary health facilities; and number of deaths in the household within the past year. Household members are those living in the same physical structure. The second half of the survey gathers information from two household members on both current and previous surgical conditions categorized into six distinct anatomical regions: face, head, and neck; chest and breast; abdomen; groin, genitals, and buttocks; back; and arms, hands, legs, and feet. Respondents answer questions based on whether they perceive themselves as ever having had a surgical condition in at least one of these anatomic regions. Additional questions cover the type of injury/accident, timing of the condition, type of health care sought, type of health care received, and reasons the individual did not access care.

Questions were modified slightly from the original SOSAS survey to better capture distinct characteristics of Nanakpur’s population. Changes were made regarding currency, transportation methods, ethnic backgrounds, and occupations. The survey was also translated into Hindi, the primary language of Nanakpur.

### Data Collection

**Figure 2:**
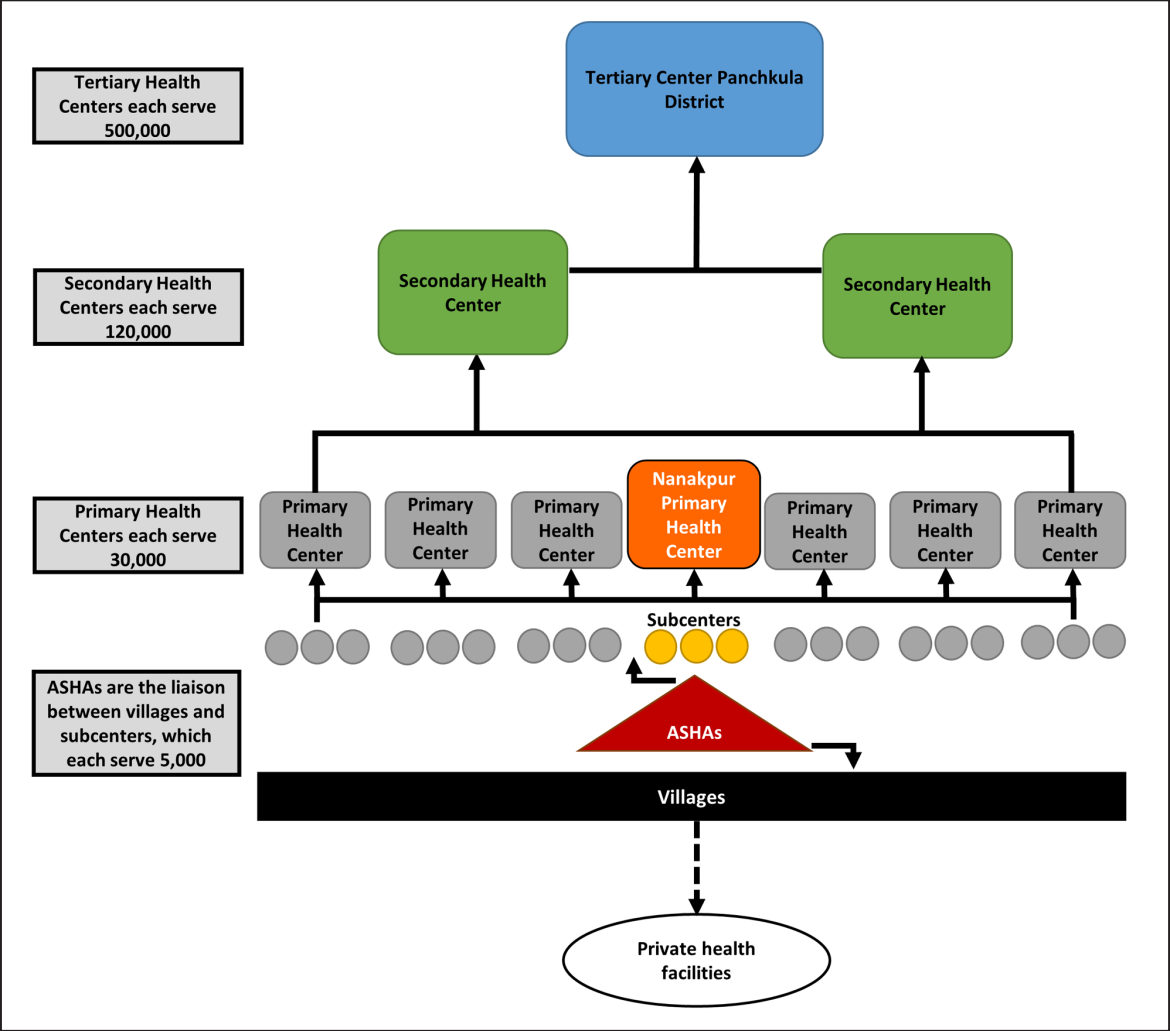
Hierarchy of health care facilities in rural India. The interaction between villages, private and public health care facilities, and ASHAs is indicated, along with the number of individuals served at each level of care [27].

India’s rural health care facilities are organized in a tiered system, from subcenters to primary, secondary, and tertiary health care centers (Figure 2) [29]. ASHAs are based in subcenters, where they provide basic preventive health services [23]. Three to five subcenters fall under each primary health center. Primary health centers serve a population of approximately 30,000, and make referrals to secondary or tertiary health centers based on the complexity of the condition [29].

The region served by the Nanakpur Primary Health Center is divided into three smaller areas served by subcenters, with each subcenter divided into eight clusters. For this study, two clusters per subcenter area were selected for sampling using a random number generator (Figure 1). Household sampling began at the geographic center of each of the six selected clusters, as determined by the ASHA with the assistance of government maps. Every fifth structure to the right of the interviewed household was approached for the survey. If there was more than one household per structure, then one household in the structure was randomly selected. If the structure was empty, then the next structure was approached. Eight to ten households were sampled in each cluster, for a total of 50 households.

Seven ASHAs participated in survey administration. Nanakpur’s lead ASHA was trained by the authors (SEC and MBB) and the Nanakpur Primary Health Center supervising physician. Topics covered included basic information on surgical capacity, the SOSAS tool, interviewing skills, and household selection methodology. The lead ASHA then trained the respective cluster’s ASHA prior to initiating survey administration in that area.

Surveys were administered in Hindi with the ASHAs as enumerators. A standardized recruitment script was read, which described the purpose of the survey and what participants would be asked to do. Verbal consent was obtained from head of the household for the first portion of the survey. After the first section of the survey was completed, verbal consent was obtained from two randomly selected household members for the second section of the survey. If a person was selected but not available for the interview, the household was revisited at a time when the household member was available. Children twelve years or older were included; consent from both the parent(s) and child were obtained. An opensource mobile data collection software (Formhub, Columbia University, 2012–2013) was used to record survey responses. Two forms, one for each portion of the survey, were created for data entry. Responses were entered at the time of survey administration, and uploaded at a later time to the data collection website. Thereafter, data were downloaded to an Excel spreadsheet (Microsoft Excel 2013 for Microsoft Office 365, Version 15.0.4833.1001. Redmond, WA: Microsoft Corporation).

### Data analysis

The following binary variables were collected: participant sex, literacy in any language, visits to a traditional healer, deaths in the household, major/minor surgical procedures, current surgical need, and unmet surgical need. *Current surgical need* was defined as a self-reported surgical problem present at the time of the interview. *Unmet surgical need* was defined as a current surgical problem for which the respondent did not access care. Education, occupation, mode of transport, availability of transport money, and reasons for not accessing care were categorical variables. Participant age, travel time, transport wait time, and cost were continuous variables. Binary and categorical variables were summarized using counts (proportions) and compared using Pearson’s chi-squared test, while continuous variables were summarized using median values with interquartile ranges (IQR) and compared using Wilcoxon rank-sum (Mann-Whitney) tests. A p-value ≤ 0.050 was considered statistically significant. Data were analyzed using STATA 13 (Stata Statistical Software: Release 13. College Station, TX: StataCorp LP).

## Results

A total of 93 individuals from 50 households participated in this study, with a 100% survey response rate. Demographic data are presented in Table 1. The median age of respondents was 35 years (IQR 26–50 years) and the median number of household members was 8 (IQR 6–9). The majority of respondents were female (86%), and many identified as either housewives (66%) or unemployed (11%). Fifty-nine percent considered themselves literate, while 38% indicated they had no education.

**Table 1:** Demographic data for surveyed individuals based on current surgical need.

Characteristic		Total population N = 93	No current need N = 28	Current need N = 65	p-value*

		N (%)			

Age (years)^†^		35 (26, 50)	33 (25, 49)	46.5 (31, 60)	0.034^§^
Sex	Female	80 (86.0)	58 (89.2)	22 (78.6)	0.174
Education	None	35 (37.6)	22 (33.8)	13 (46.4)	0.251
	Some	58 (62.4)	43 (66.1)	15 (53.6)	
Literacy	Yes	55 (59.1)	40 (61.5)	15 (53.6)	0.473
Occupation	Unemployed/Homemaker	71 (76.3)	51 (78.5)	20 (71.4)	0.464
	Employed outside	22 (23.7)	14 (21.5)	8 (28.6)	

* Current need vs. no current need using Pearson’s chi-squared test. ^†^ Median (IQR). ^§^ Kruskal-Wallis test.

Cost and duration of transport to health care centers differed by level of facility. Median cost of transport in rupees (INR) to tertiary centers was greater compared to primary centers (27.5 INR, IQR 0–35 INR vs. 10 INR, IQR 0–10 INR), and median travel time in minutes to tertiary centers was greater compared to secondary centers (60 min, IQR 45–90 min vs. 45 min, IQR 30–60 min) and primary centers (30 min, IQR 15–60 min). Eighteen individuals (20.9%) indicated they did not utilize primary health centers, 16 individuals (18.8%) did not utilize secondary centers, and 33 individuals (37.1%) did not utilize tertiary centers, instead preferring to seek care from private health facilities.

Twenty-five (50.0%) participants reported a history of a having undergone a surgical procedure, of which 17 were major (requiring regional or general anesthesia) and eight were minor (dressings, wound care, punctures, suturing, or incision and drainage). Twenty-eight individuals (30.1%) indicated they had a current surgical need at the time of the interview, the majority of which were musculoskeletal (Table 2). The underlying pathology between those with a current surgical need versus those without one showed a majority of the conditions for those without a current surgical need were secondary to injuries N = 10 (50.0%) or a growth/mass N = 4 (20.0%), while a majority of conditions for those with a current need were wounds by injuries N = 7 (31.8%), congenital deformities N = 7 (31.8%), and acquired deformities N = 7 (31.8%), p = 0.035. Nine of the current need conditions were injuries or accidents due to a motorcycle crash (N = 1, 3.6%), stab/slash wound (N = 1, 3.6%), household incident (N = 2, 7.1%), fall (N = 4, 14.3%), or burn (N = 1, 3.6%). Nineteen subjects (67.9%) reported that their current surgical need was not due to an injury or accident. Compared to those without a current surgical need, those with one were older (46.5 vs. 33 years, p = 0.034) and more likely to lack funds to travel to a tertiary center (64.3% vs. 32.4%, p = 0.041). The most frequent disability reported among individuals with a current surgical need was inability to work (N = 17). Other demographic characteristics comparing individuals with and without current surgical need are listed in Table 1.

**Table 2:** Anatomic location of conditions with current surgical need.

Anatomic location	N (%)

Face, head, neck	2 (7)
Chest	1 (4)
Back	1 (4)
Abdomen	3 (11)
Groin	4 (14)
Extremities	17 (60)
TOTAL	28 (100)

Analysis of variables that may impact access to surgical care demonstrated that transport time and costs generally increased as facility level rose. In particular, median costs of travel to health facilities for individuals with a current surgical need were estimated to be either the same or greater as compared to individuals without a current need (Table 3).

**Table 3:** Predictors for accessing surgical care by facility level and current surgical need.

Facility level	Access variable		No current need	Current need	p-value*

			N (%)		

Primary	Transport type	On foot/carried	7 (11.3)	2 (8.0)	0.313
		Personal	21 (33.9)	4 (16.0)	
		Public	22 (35.5)	13 (52.0)	
		Don’t go	12 (19.4)	6 (24.0)	
	Travel time (min)^†^		30 (15, 60)	30 (15, 60)	0.873^§^
	Transport wait time (min)^†^		0 (0, 15)	0 (0, 15)	0.966^§^
	Cost (Indian rupee)^†^		0 (0, 10)	10 (0, 10)	0.182^§^
	Availability of	Yes/NA	34 (85.0)	14 (77.8)	0.501
	transport money	No	6 (15.0)	4 (22.2)	

Secondary	Transport type	On foot/carried	0 (0.0)	0 (0.0)	0.284
		Personal	18 (30.0)	4 (16.0)	
		Public	30 (50.0)	17 (68.0)	
		Don’t go	12 (20.0)	4 (16.0)	
	Travel time (min)^†^		30 (30, 60)	60 (30, 60)	0.0495^§^
	Transport wait time (min)^†^		15 (0, 30)	15 (0, 30)	0.929^§^
	Cost (Indian rupee)^†^		20 (0, 25)	20 (20, 20)	0.730^§^
	Availability of	Yes/NA	29 (78.4)	14 (77.8)	0.960
	transport money	No	8 (21.6)	4 (22.2)	

Tertiary	Transport type	On foot/carried	2 (3.2)	0 (0.0)	0.225
		Personal	20 (31.8)	4 (15.4)	
		Public	18 (28.6)	12 (46.2)	
		Don’t go	23 (36.5)	10 (38.5)	
	Travel time (min)^†^		60 (45, 60)	90 (60, 150)	0.0626^§^
	Transport wait time (min)^†^		5 (0, 30)	12.5 (0, 45)	0.742^§^
	Cost (Indian rupee)^†^		10 (0, 35)	30 (0, 35)	0.305^§^
	Availability of	Yes/NA	29 (85.3)	13 (92.9)	0.471
	transport money	No	5 (14.7)	1 (7.1)	

* Current need vs. no current need using Pearson’s chi-squared test. ^†^ Median (IQR). ^§^ Kruskal-Wallis test.

Out of the 28 individuals with a current surgical condition, six had not accessed care and were thus categorized as having an unmet surgical need (6.5%; 95% CI 2.45–13.5%). Two respondents cited lack of funds as the reason for not accessing surgical care; one respondent each cited other reasons: lack of time; lack of necessary health care personnel, facility, or equipment; or did not perceive a need. One respondent did not provide a reason for not seeking care. Six deaths were reported in the previous year; one death was perceived by the respondent to be due to cost of transportation for accessing surgical care. The other five households indicated no perceived need for surgical care prior to their family members’ deaths; deaths were due to various reasons, including cardiac conditions and infections.

## Discussion

The first implementation of the SOSAS survey in India is described in this Nanakpur pilot study, which provides preliminary population-based data on the burden of surgical disease in India, and contributes to our understanding of the epidemiology of surgical diseases in LMICs. In addition, the study assesses the feasibility of utilizing the ASHA community health worker system as enumerators for population-based studies, such as the SOSAS survey.

Similar to other LMICs, including Nepal, rates of current and unmet surgical need in Nanakpur are high [6,11,12,13,30]. Nepal and India have much in common, including the challenges of limited access to health care in rural settings [31,32]. Compared with the countrywide Nepal SOSAS study, these data from Nanakpur demonstrate a lower unmet surgical need of 6.5% compared to Nepal’s 10.0% [6]. This difference in unmet surgical need is multifactorial. Varying sample compositions likely contributed, as the Nepal study may have included communities with more barriers to surgical care than Nanakpur. India also has a greater number of physicians per 1000 individuals than Nepal (0.7 vs. 0.1) [33]. However, such comparisons between India and other LMICs would be better characterized with a countrywide India SOSAS study.

The survey further revealed that Nanakpur’s community members recognize multiple barriers to accessing surgical care. Each year, between October and June, a migrant brick laborer population travels to Nanakpur to work at one of several factories located throughout the area. As income is determined on a day-to-day basis, these brick laborers anecdotally described financial constraints and long wait times in government hospitals as barriers to seeking health care. Among the six individuals with an unmet surgical need, the brick laborer (N = 1) stated that he did not access care due to a lack of money for transport. Five villagers had an unmet surgical need, of whom four provided reasons for not seeking medical attention: lack of funds (N = 1), lack of time (N = 1), lack of adequate health care facilities (N = 1), and lack of perceived need for care (N = 1).

Many of these barriers to accessing surgical care could be addressed with the further development of surgical capacity at lower level facilities, as well as a more efficient referral system [34,35]. Dare et al. demonstrated that being less than 50 kilometers from a well-equipped district hospital in India reduces the possibility of mortality from acute abdominal conditions by two-thirds [21]. Thus, the delivery of safe surgical and anesthesia care in Nanakpur is contingent on developing the surgical capacity of secondary and tertiary centers, which are supposed to provide operative care for the treatment of common surgical and obstetrical conditions per the Government of India [21]. Increasing the availability of medications, equipment, supplies, and banked blood at the primary and secondary health centers are crucial steps as well [2,35]. This enables adequate care for basic surgical problems at lower level hospitals, as well as the opportunity to stabilize more critically ill patients prior to transport to tertiary centers for more complex procedures.

In addition to health care facility capacity development, community-based educational programs for strengthening prehospital systems are essential for targeting cited barriers to accessing medical care. Extremities were found to be the predominant anatomical region of current surgical need (N = 17); five extremity conditions were from injuries, of which two were caused by falls secondary to respondents’ physically demanding tasks as farmers and laborers. The implementation of first responder courses in Nanakpur could address this issue by training laypeople to perform initial stabilization and transportation of the injured to a higher level of care [36]. These courses cover topics including cardiopulmonary resuscitation, fracture management, and triage [37,38]. Such programs have been shown to decrease mortality and physiological severity scores in trauma patients, and can improve prehospital infrastructure by empowering community members with basic emergency and trauma care skills [36].

It is broadly accepted that global surgical initiatives are best performed with the commitment, desire, and participation of the local population [2,39]. In previous SOSAS studies, native medical and nursing students have been trained as enumerators [6,11,12]. Our study demonstrates the utility of ASHAs for conducting SOSAS surveys, which suggests a reliable methodology for performing a countrywide SOSAS study. Moreover, the ASHA system could serve as a foundation for identifying and mitigating barriers to accessing surgical care in rural India. The ASHAs are a well-known and accepted presence within rural Indian communities, familiar with village families, and able to speak the local language and dialect. Given their proximity to the local population and demonstrated investment in villagers’ health, the ASHAs would likely be adept first responders [36,37,38]. The ASHAs could also be trained to elicit symptoms, perform physical examinations, and assist in the detection of early surgical site infections, as they are accustomed to making daily trips to villagers’ homes for maternal and child health assessments [40]. Such training and follow-up could occur via telemedicine with surgeons at tertiary hospitals. ASHAs work closely with health care staff at primary and community health centers, allowing for physician interaction and relationship building, thus promoting the clinicians to trust the ASHAs’ judgment.

There are a number of limitations to this study. First, the sample of 93 respondents was small and findings are likely not generalizable to other areas of the country, as India has a diverse population with varied cultures, languages, climates, and landscapes. In addition, results were based on individuals’ self-reported conditions and were not consistently confirmed through review of records, posing the issue of recall bias as well as the potential for inaccurate estimation of surgical conditions, as some self-diagnoses may not qualify as surgical if evaluated by a medical professional. Some respondents did not provide responses to all questions resulting in missing data. Respondents may have modified their answers because of the presence of foreign medical students who were not community members themselves and lacked a pre-existing level of trust and connectedness with the interviewees, which is highly valued in these communities. ASHAs assisted with the translation of answers, but it is possible that some details were lost in this process. Future SOSAS studies could include confirmation with medical records in order to validate verbal responses, and address the issues of recall, cultural, diagnostic, and translational biases.

Medical student enumerators initially attempted visual physical examinations, similar to those conducted by Gupta et al.; however, these were ultimately excluded out of respect for local sensibilities and cultural standards [6]. Evaluations by resident and attending physicians were unable to be incorporated due to staffing limitations. Furthermore, enumerators only surveyed individuals who were present at the time of the interview; since visits were made during the morning and afternoon due to logistical issues of transportation availability and safety concerns, most males were at work, making a significant portion of household members interviewed female. World Health Organization data shows that the number of unintentional injuries in LMICs is greater in males, so the number of surgical conditions secondary to injuries found in our study is likely an underestimation [41]. Finally, by defining unmet surgical need as individuals who have a current need but have not sought health care, it is assumed that those who did seek care received appropriate treatment. However, there may have been individuals who received care at the primary health center but required a higher level of care, and could not access it due to factors such as financial or transportation constraints. Thus, this suggests that the unmet need of 6.5% found in this study is a conservative estimate of the true unmet need.

## Conclusions

Nanakpur is the first area in India where the SOSAS survey was implemented, which suggests a significant burden of current and unmet surgical need. A countrywide SOSAS initiative could address this pilot study’s limitations by improving interview timing and including objective confirmatory measures to correct biases. Involvement of ASHAs as done in this study is one method to help overcome many logistical and sociocultural barriers presented by the heterogeneity of the Indian population. Accordingly, the ASHA system is a constant throughout India that can be employed for a countrywide SOSAS study, as well as other future initiatives to develop surgical capacity. Findings from this study will also inform the execution of basic, community-level first responder courses in Nanakpur, and highlight the need to further evaluate additional effective interventions to strengthen surgical systems in rural India.
